# Cutaneous Squamous Cell Carcinoma Risk Factors: Are Current Criteria Still Valid? A Retrospective, Monocenter Analysis

**DOI:** 10.3390/life15081257

**Published:** 2025-08-07

**Authors:** Maike Kaufhold, Sepideh Asadi, Yalda Ghoreishi, Annika Brekner, Stephan Grabbe, Henner Stege, Hadrian Nassabi

**Affiliations:** Department of Dermatology, University Hospital Mainz, 55122 Mainz, Germany; sasadi@uni-mainz.de (S.A.); yalda.ghoreishi@unimedizin-mainz.de (Y.G.); annika.brekner@unimedizin-mainz.de (A.B.); stephan.grabbe@unimedizin-mainz.de (S.G.); henner.stege@unimedizin-mainz.de (H.S.); hadrian.nassabi@srh.de (H.N.)

**Keywords:** squamous cell carcinoma, real-world data

## Abstract

Introduction: Cutaneous squamous cell carcinoma (cSCC) is the second most common skin cancer entity in Germany, following basal cell carcinoma. Its incidence has increased fourfold over the past three decades. Early diagnosis and treatment are essential for achieving favorable outcomes. Our study aims to identify prognostic factors based on real-world data to improve follow-up protocols and raise clinical vigilance. Methods: We conducted a retrospective, monocenter analysis with a total of 124 patients with at least one cSCC thicker than 3 mm, treated at the Department of Dermatology, University Medical Center Mainz, between 2010 and 2020. Tumor-specific criteria were correlated with patient-specific data, such as gender, age, immunosuppression, UV exposure and mortality. Results: A higher incidence of cSCC was found on UV-exposed skin (91.1%); however, tumors on non-UV-exposed skin were on average thicker (6.55 mm vs. 9.25 mm, ***p* = 0.011**) and associated with higher metastasis rates (10.6% vs. 63.3%, ***p* < 0.001**). Immunosuppression was strongly associated with a younger age at diagnosis (74 years vs. 81 years), a higher metastasis rate (29% vs. 10.8%, ***p* = 0.021**) and a worse 5Y-OS-rate (36.1% vs. 97.8%, ***p* = 0.04**). SLNB was performed in eight patients, with one positive SLN identified (12.5%). Local recurrence was observed in 18.1% (*n* = 21) of patients who did not experience SLNB, whereas no local recurrences (0%) were reported in patients with SLNB (*p* = 0.349). Discussion: Tumors on non-UV-exposed areas were thicker and more often metastatic, suggesting delayed detection or more aggressive tumor subtypes. Immunosuppression was associated with worse outcomes, underscoring the need for intensified follow-up. SLNB was rarely performed, and larger studies are needed to assess its role.

## 1. Introduction

Cutaneous squamous cell carcinoma (cSCC) is the second most common type of skin cancer, predominantly affecting the elderly population. The incidence in Germany has recently increased rapidly and is expected to double by 2030 [1]. With early diagnosis and treatment, the prognosis is generally favorable; however, local recurrences or metastases significantly worsen the outcome. Despite the existence of the current S3 guideline with numerous therapy recommendations and standards, controversial discussions persist.

The objective of this study is to evaluate risk factors based on clinical real-world data to improve follow-up protocols and raise clinical vigilance. Several clinical and histopathological features, such as perineural invasion; tumor location on the lip, temple or ear; and immunosuppression, have been identified as adverse prognostic factors in cutaneous squamous cell carcinoma. These parameters will be specifically addressed in our analysis. In addition, this study will also address the controversially discussed diagnostic procedure of sentinel lymph node biopsy. While recent studies, such as those by Tejera-Vaquerizo et al. [2] and Zhang et al. [3], suggest a potential survival benefit, other analyses, e.g., by Kofler et al. [4], have not confirmed a significant advantage.

Given the limited sample sizes and the dated nature of many existing studies, we see a need to re-evaluate established risk factors and tumor characteristics in a contemporary, real-world patient cohort. The analysis includes data from 124 patients treated at the Department of Dermatology, University Medical Center Mainz, between 2010 and 2020.

## 2. Methods

The data of the 124 patients evaluated were retrieved from the patient management system SAP (Cerner Health Services, Inc., 51 Valley Stream Parkway, Malvern, PA 19355, USA). Patients were included if they met the following criteria: a histologically confirmed diagnosis of cutaneous squamous cell carcinoma with a tumor thickness greater than 3 mm, regular follow-up care at the University Hospital of Mainz and had sufficient clinical documentation available. Patients were excluded if the tumor thickness was less than 3 mm, if clinical documentation was insufficient or if follow-up was conducted outside the study center. The following patient data were collected: date of birth, gender, date of initial diagnosis, age at the time of initial diagnosis, date of last follow-up, immunosuppression, local recurrence, metastasis (lymph node metastasis, distant metastasis), sentinel lymph node biopsy (SLNB), follow-up duration and mortality status. Since there is currently no formal recommendation for SLNB in national cSCC guidelines, SLNB was not performed based on standardized selection criteria at our institution. The decision to perform SLNB was made individually by the treating physician, depending on clinical judgment and patient-specific factors. As a result, SLNB was performed in a non-systematic, retrospective manner. Furthermore, a comprehensive set of tumor parameters was recorded, including tumor thickness, tumor location, UV-exposed location, R-status, perineural invasion, the occurrence of multiple cSCCs and the number of cSCCs with a thickness greater than 3 mm. In cases of multiple tumors, the thickest tumor was selected for analysis.

The follow-up was conducted retrospectively, spanning from the initial date of diagnosis and concluding at the last documented clinical contact or the patient’s death. The median follow-up duration was 51 months (IQR 46.0–57.5 months). Patients were examined dermatologically every 3 to 12 months in accordance with the national guideline for cSCC. Missing data primarily resulted from incomplete or insufficient clinical documentation in the patient records. Affected cases were excluded from the respective analyses, and the reduced sample size (*n*) was reported accordingly for each variable. No data imputation was applied.

A statistical analysis was performed using IBM SPSS Statistics Version 27 for Windows and was supported by the Institute for Medical Biometry, Epidemiology, and Informatics (IMBEI) at the University Medical Center Mainz. Given their non-normal distribution, quantitative variables such as age and tumor thickness are reported as medians with interquartile ranges (IQRs). Categorical variables, such as location, UV exposure and gender, were calculated as frequencies. Associations were evaluated using Pearson’s chi-squared test or Fisher’s exact test for analyses with small sample sizes. Survival statistics were analyzed using Kaplan–Meier survival analysis and the log-rank test. A *p*-value of ***<0.05*** was considered statistically significant. Due to the limited number of events, such as metastases and local tumor recurrence, we refrained from conducting multivariate analyses. Including multiple covariates in the statistical model would have led to overfitting and potentially unreliable conclusions.

### 2.1. Definition of Variables

#### 2.1.1. Definition of UV-Exposed Skin

The head, neck, décolleté, forearms, hands and lower legs were classified as UV-exposed skin. In contrast, tumors on the trunk, upper arms, thighs and feet were categorized as non-UV-exposed.

#### 2.1.2. Definition of Immunosuppression

Patients were classified as immunosuppressed if they had a history of organ transplantation, chronic lymphocytic leukemia (CLL), myeloproliferative syndrome (MPS), non-Hodgkin lymphoma (NHL) or infection with Human Immunodeficiency Virus (HIV). Additionally, patients were deemed to be immunosuppressed if they had been treated for an extended period with immunosuppressive medications, such as glucocorticoids or methotrexate.

#### 2.1.3. Definition of R-Status

The R-classification is used to describe the status of residual tumor after a surgical resection [5]. Rx means that the residual tumor cannot be assessed. R0 indicates tumor-free resection margins, R1 represents microscopic residual tumor and R2 refers to macroscopic residual tumor.

## 3. Results

### 3.1. Descriptive Analysis

A total of 124 patients were included in this study, each with at least one cSCC with a tumor depth of ≥3 mm. Multiple tumors were identified in 35 patients (28.2%), with two tumors being the most frequently observed number (*n* = 14, 11.3%). The cohort comprised 33 women (26.6%) and 91 men (73.4%), and the median age was 79 years (IQR: 72–85 years).

The distribution of tumor locations was as follows:

o Head and neck: 89 cases (71.8%).
o Legs: 17 cases (13.7%).
o Arms: 13 cases (10.5%).
o Trunk: 5 cases (4%).

When analyzing tumor thickness by anatomical region, tumors on the trunk exhibited the greatest median thickness, measuring 15.0 mm (IQR: 10.0–20.0 mm). In comparison, tumors on the head (median: 5.6 mm; IQR: 3.6–7.6 mm), arms (median: 3.95 mm; IQR: 2.0–6.95 mm) and legs (median: 6.0 mm; IQR: 3.75–8.25 mm) were significantly thinner than those on the trunk (***p* = 0.018**).

Of the analyzed tumors, 113 (91.1%) were located on UV-exposed skin. Tumors located on non-UV-exposed areas accounted for 11 cases (8.9%). These findings highlight the predominance of cSCC in UV-exposed areas, particularly the head and neck region. The average tumor thickness varied significantly between UV-exposed and non-UV-exposed skin. On UV-exposed areas, the median tumor thickness was 5.4 mm (IQR: 3.55–7.25 mm), while tumors on non-UV-exposed areas showed a significantly greater median thickness of 8.0 mm (IQR: 6.0–10.0 mm) (***p* = 0.011**).

**Figure 1:**
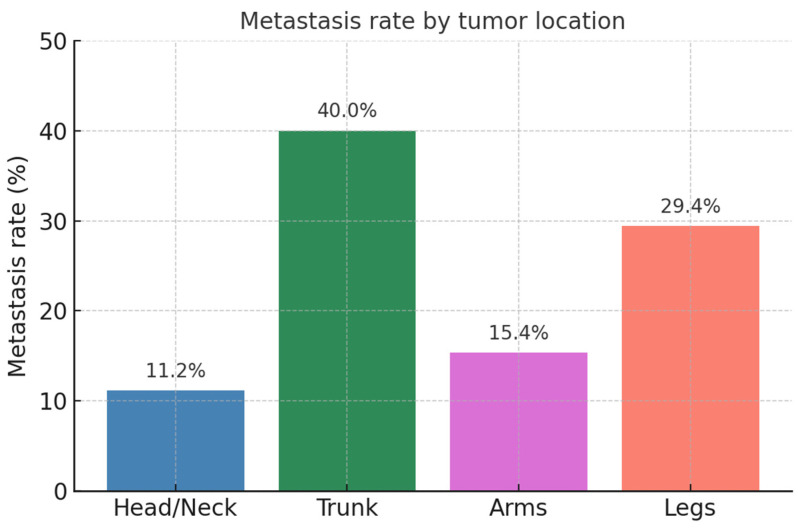
Metastasis rate by tumor location.

Out of the 124 patients included in this study, 19 (15.3%) exhibited metastases, with 17 cases (13.7%) manifesting as lymph node metastases and 4 cases (3.2%) as distant metastases. Tumors located on UV-exposed areas had a metastasis rate of 10.6% (*n* = 12), whereas non-UV-exposed tumors demonstrated a markedly higher metastasis rate of 63.6% (*n* = 7; ***p* < 0.001**, Figure 1).

Tumor thickness was found to be a significant predictor of metastasis. Tumors with a thickness of 3.0 mm to 5.9 mm (*n* = 64) had a metastasis rate of 3.1% (*n* = 2), while tumors thicker than 6 mm (*n* = 57) showed a considerably higher metastasis rate of 26.3% (*n* = 15; ***p* < 0.001**).

Notably, 15 patients (83.3%) developed metastases within the first 24 months after diagnosis. Seven patients developed metastases within 6 months of the initial diagnosis, eight patients between 7 and 24 months and one patient after 24 months. In two patients, the date of metastasis could not be determined.

Local tumor recurrence (histologically confirmed) occurred in 21 patients (16.9%). In 80.9% of patients (*n* = 17), local recurrences occurred within the first two years after the initial diagnosis (median 9.5 months, IQR: 5.0–16.5 months). Among recurrent tumors, the median thickness was 5 mm (IQR 2.5–7.5 mm). Out of 120 patients, excluding 4 due to missing data, 60 underwent re-excision. Re-excision was performed in cases where the initial histopathological report indicated an incomplete tumor removal. Among those, 25% (*n* = 15) experienced a local recurrence. In contrast, only 4 out of 60 patients (6.67%) who did not experience re-excision developed a local recurrence (***p* = 0.006**). Perineural invasion was detected in 4 out of 123 patients (3.25%). Among these, three (75%) developed recurrence. Compared to patients without perineural invasion, the risk of recurrence was 5.25 times higher (***p* = 0.013**).

All patients had their tumor surgically removed (*n* = 124, 100%). In 117 patients (94.35%), the tumor was completely removed through resection (R0); in 6 patients (4.8%), residual tumor lesions were present after resection (R1); and in 1 patient (0.8%), macroscopic tumor residuals were identified (R2). The analysis of resection margins revealed differences in metastases and local recurrence rates; however this was not statistically significant. Among patients with R0 resection margins, 13.8% (*n* = 16) developed metastases, and 15.5% (*n* = 18) experienced local recurrence. In contrast, patients with R1 resection margins showed higher rates, with 50% (*n* = 3) developing metastases and 33.3% (*n* = 2) experiencing local recurrence. The results did not reach statistical significance (metastases: *p* = 0.052; local recurrence: *p* = 0.446).

When analyzing overall survival (OS), the four-year survival rate was 77.1% in patients with a tumor thickness greater than 6 mm, compared to 61.5% in patients with a tumor thickness between 3 and 5.9 mm (*p* = 0.525). Thus, we were unable to demonstrate increased mortality in those with tumors with a thickness greater than 6 mm.

Table 1 gives a comprehensive overview of the variables.

### 3.2. Immunosuppression

Out of 124 patients included in this study, 31 (25%) were taking immunosuppressive medication. The patient characteristics are shown in Table 2. Immunosuppressed cSCC patients were significantly younger at the time of diagnosis compared to non-immunosuppressed patients (***p* = <0.001**). The median age at diagnosis for non-immunosuppressed patients was 81 years (IQR: 75.5–86.5 years), while immunosuppressed patients had a median age of 74 years (IQR 70.5–77.5 years). Patients with a history of organ transplantation had a median age of 67.5 years at the time of initial diagnosis (IQR 59.0–76.0 years). In contrast, patients without organ transplantation had a significantly higher median age of 80 years at initial diagnosis (IQR 75.5–85.5 years). This resulted in a significant age difference between the two groups (***p* < 0.001**).

Regarding tumor location, we observed comparable patterns in patients receiving immunosuppressive medication and those who are immunocompetent. Tumors were most often found on UV-exposed skin (83.9%; *n* = 26).

In 64.5% (*n* = 20) of immunosuppressed patients, tumors were located in the head and neck region. In terms of tumor thickness, the median tumor thickness was identical in both groups at 5.6 mm.

Immunosuppressed patients demonstrated a higher likelihood of developing multiple cSCCs compared to non-immunosuppressed patients (38.7%; *n* = 12 vs. 24.7%; *n* = 23), although this difference was not statistically significant (*p* = 0.186). Local recurrence was observed in 25.8% (*n* = 8) of immunosuppressed patients compared to 14% (*n* = 13) of non-immunosuppressed patients, which also did not reach statistical significance (*p* = 0.166). Metastases, however, occurred significantly more frequently in immunosuppressed patients, with a rate of 29% (*n* = 9) compared to 10.8% (*n* = 10) in non-immunosuppressed patients (***p* = 0.021**).

**Figure 2:**
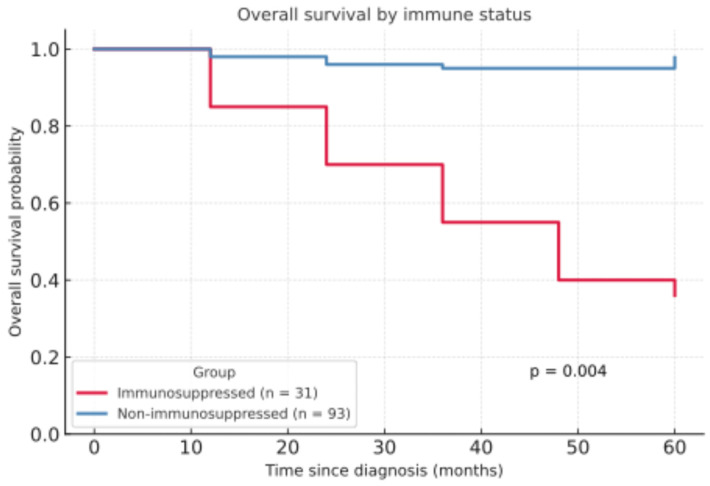
Overall survival by immune status.

OS was significantly lower among immunosuppressed patients (Figure 2). The five-year overall survival rate (5Y-OS) was 36.1% in immunosuppressed patients compared to 97.8% in non-immunosuppressed patients (***p* = 0.004**). Regarding the follow-up period, patients taking immunosuppressive medication were monitored for an average of 24.79 months, while immunocompetent patients had a significantly shorter average follow-up period of 14.97 months (***p* = 0.013**).

### 3.3. Sentinel Lymph Node Biopsy

The decision to perform SLNB was made on a case-by-case basis, as it is not currently included in clinical guidelines for cutaneous squamous cell carcinoma. No standardized criteria were applied. In the study cohort of 124 patients, SLNB was performed in 8 patients, with one positive SLN identified (12.5%). Tumor thickness was notably greater in patients who underwent SLNB (median 11 mm, IQR 5.56–16.88 mm), compared to a median of 5.4 mm (IQR 3.4–7.4 mm) in patients without SLNB (***p* = 0.011**). The SLNB group was significantly younger than those who did not receive an SLNB (median 72.5 years, IQR 61.25–78.75 years vs. median 80 years, IQR 74 years–85 years; ***p* = 0.038**).

Local recurrence was observed in 18.1% (*n* = 21) of patients who did not experience SLNB, whereas no local recurrences (0%) were reported in patients with SLNB. However, this difference was not statistically significant (*p* = 0.349).

Metastases occurred in 13.8% (*n* = 16) of patients without SLNB, compared to 25% (*n* = 2) of those who underwent SLNB. Nevertheless, the difference was not statistically significant (*p* = 0.328). One out of two patients who developed metastases showed a positive SLNB in advance. The second patient, who developed lung metastases, showed a negative SLN.

### 3.4. Development of Metastases

Out of a total of 124 patients, 15.3% (*n* = 19) developed metastases. Among these, 14 patients (11.29%) developed lymph node metastases only, 3 patients (2.42%) developed both lymph node and distant metastases, 1 patient (0.81%) developed distant metastases only and 1 patient developed cutaneous metastases only (0.81%). Tumor thickness and patient-specific factors were significantly associated with metastasis risk. The median tumor thickness in patients without metastases was 5 mm (IQR 3.9–7.5 mm), whereas in patients with metastases, the median thickness in patients with metastases was 8.0 mm (IQR 6.25–10.5 mm; ***p* < 0.001**). Tumors located on UV-exposed areas demonstrated a lower likelihood of metastasis compared to those in non-UV-exposed regions.

Immunosuppression was also strongly correlated with metastasis. Among patients with metastases, 47.4% (*n* = 9) were immunosuppressed, compared to 21.0% (*n* = 22) in patients without metastases (***p* = 0.021**).

**Figure 3:**
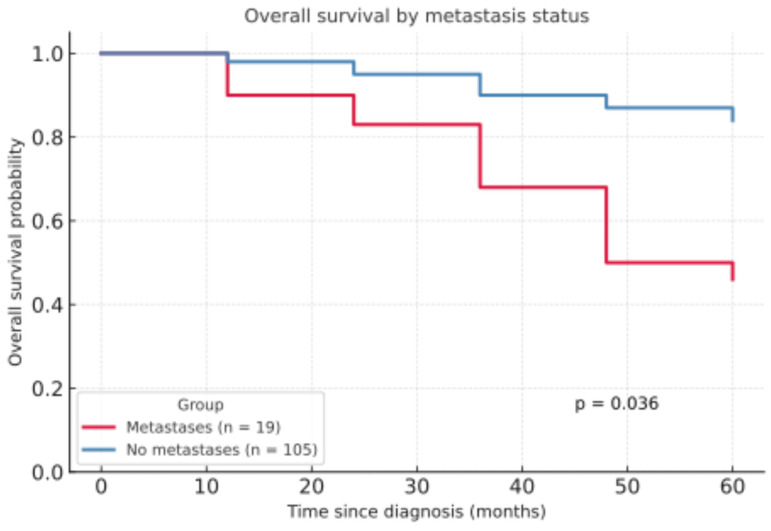
Overall survival by metastasis status.

The development of metastases had a notable impact on 5Y-OS. Patients without metastases had a significantly higher 5Y-OS rate of 83.9%, compared to 46.1% in those with metastases (***p* = 0.036**, Figure 3).

## 4. Discussion

The aim of the study was to conduct a retrospective analysis of real-world data from 124 patients with cSCC over a period of 10 years at the Department of Dermatology, University Medical Center Mainz, and to compare our findings with the current literature.

### 4.1. Descriptive Analysis

A comparative analysis of the data revealed that the prevalence of cSCC was higher in the male subjects (*n* = 91) compared to the female subjects (*n* = 33), which aligned with the current epidemiological trend [6,7,8]. The average age was 77.74 years, slightly older than that in the observations made by Rudolph et al. (74.8 ± 10.7 years) [9], Harris et al. (70.4 years) [7] and Brantsch et al. (72 years) [6].

The majority of cSCCs in our cohort occurred on UV-exposed skin (91.1%, *n* = 113), with most tumors located in the head and neck region (71.8%, *n* = 89). These findings were consistent with the observations made in a retrospective analysis of 194 patients by Mourouzis et al. [10] and by Brantsch et al. with 80% of cSCCs located in the head and neck region [6]. A major risk factor for the development of cSCC is cumulative UV exposure, which leads to DNA damage [11]. Additionally, in older individuals, reduced DNA repair leads to abnormal cell growth and more mutations [12]. Therefore, it is not surprising that cutaneous squamous cell carcinoma predominantly occurs at UV-exposed sites and tends to be more common in older patients. The fact that in the present study, tumors on non-UV-exposed skin showed higher tumor thickness than on UV-exposed skin is not consistent with a recent publication by Morello-Vicente et al., who could not show significant differences in tumor depth between UV- and non-UV-exposed skin [13]. Also, our study revealed a significant increase in metastasis for tumors located on non-UV-exposed skin. This observation does not align with the current literature, which predominantly indicates that location on the ear, temple or lip—thus in UV-exposed areas—is associated with an increased risk of metastasis [14,15,16,17] and local recurrence [13]. The higher metastasis rate in tumors on non-UV-exposed skin may be explained by their greater thickness, likely due to delayed detection or more aggressive tumor behavior. Due to the very limited number of cases within each specific subregion of the head and neck area, we refrained from a more detailed statistical analysis of the subregions.

Overall, the metastasis rate in this study (15.3%) is comparable to the metastasis rate from 0.5% to 20.7% reported in the literature [6,15,16,18,19,20,21]. Tumor thickness is a known risk factor for the development of metastases and local recurrence [6,21,22], which is reflected in our data.

Among patients who developed metastases, the majority (83.3%) did so within the first 24 months of follow-up. In a study conducted by Krediet et al., 143 patients were analyzed regarding the occurrence of metastases, finding that all metastases occurred within 24 months of follow-up. As risk factors, recurrent disease or tumor thickness > 4 mm were identified [23]. This would imply that a 2-year period is crucial for sufficient follow-up.

Our study demonstrated a local recurrence rate of 16.9% (*n* = 21), which is slightly higher compared to the rates reported in the literature [24]. Most recurrences occurred in patients with primary tumors located in the head and neck region (71.4%, *n* = 15), a region at high risk for local recurrence as stated by Harris et al. [7]. In further studies, local recurrences occurred in 75% of cases within two years and in 83% of cases within three years after the initial diagnosis. No recurrences were observed more than six years after the primary diagnosis [6,14]. The German guideline on actinic keratosis and cSCC recommends closer follow-up during the first two years after the primary diagnosis. In high-risk patients, an ultrasound of the regional lymph nodes is advised during the first two years of follow-up to monitor for metastasis [25,26,27,28,29]. Our data is therefore in line with the current guideline recommendation.

In our study, the rate of incomplete excisions (R1) was 5.7%. Compared to a retrospective study by Svensson et al. which reported an 11.7% rate of incomplete excisions in 691 cSCC patients, our study demonstrated a lower rate. In contrast to our study, Svensson et al., Stewart et al. and Ribero et al. reported an increased rate of incomplete excisions in tumors located in the head and neck region [30,31,32]. Incomplete resection margins have been identified in the literature as significant risk factors for the development of regional lymph node metastases [10,33] and local recurrences [27,34]. In the present study, no significant association between R-status and the development of metastases or local recurrences could be established (metastases: *p* = 0.052; local recurrence: *p* = 0.446).

Our results did not demonstrate an increased mortality rate for tumors with a thickness greater than 6 mm. The 5Y-OS rate was 72.2%, and that for 3Y-OS was 93.8%. However, some studies have shown a correlation between tumor thickness and survival [35,36,37]. Friedmann et al. reported that all tumors with a fatal outcome were at least 10 mm thick and extended to the subcutaneous tissue and deeper structures [37]. Potential causes for the discrepancies between our survival data and the current literature could be the comparatively short follow-up period (17.32-month average) or the continuation of follow-up care by local dermatologists, leading to the potential loss of follow-up at our site. Therefore, it is not possible to exclude the existence of a higher mortality rate in our cohort than that which we were able to document.

### 4.2. Immunosuppression

Immunosuppression is a well-known risk factor for the development of cSCC. We included patients with various causes of immunosuppression, which limits the interpretability and generalizability of our findings. In our study, it was observed that patients with existing immunosuppression were significantly younger at the time of diagnosis compared to patients without immunosuppression, especially those with a history of prior organ transplantation. Our observations are consistent with the findings from Harwood et al., who reported that transplant patients were, on average, 15 years younger at the time of cSCC diagnosis compared to immunocompetent individuals [38].

Immunosuppressed patients are more susceptible to the damaging effects of UV radiation and pro-carcinogenic viruses [39]. Thus, cSCCs in immunosuppressed patients are classified as more aggressive high-risk cSCCs [40]. Unlike low-risk squamous cell carcinomas, regular sonographic examinations and, if necessary, advanced imaging are suggested during the first two years of follow-up [29]. This recommendation is also reflected in our study cohort, where immunosuppressed patients were monitored for a significantly longer period compared to patients without immunosuppression (24.79 month vs. 14.97 months, ***p* = 0.013**).

A significant difference in survival and metastasis rates was observed between patients with and without immunosuppression. In our study, patients with immunosuppression developed more metastases (29%, *n* = 9) compared to immunocompetent patients (10.8%, *n* = 10; ***p* = 0.021**). The same results were found by Genders et al. and De Jong et al. [41,42]. Additionally, out cohort showed a significantly lower five-year survival rate in immunosuppressed patients (36.1%) compared to immunocompetent patients (97.8%; ***p* = 0.004**). The less favorable prognosis regarding survival in immunosuppressed patients is reflected in the literature [43,44,45]. No significant difference in our cohort was found regarding local recurrence rates, which does not support the results of Gonzalez et al. and Lopez et al. [46,47]. For future analyses with larger cohorts, it would be of particular interest to differentiate between the underlying cause of immunosuppression, to assess CD4+ T-cell counts in HIV-positive patients and to look at the specific immunosuppressive agents used.

### 4.3. Sentinel Lymph Node Biopsy

The sentinel lymph node is usually the first lymph node to be affected by metastasis from a primary tumor [48]. Metastasis in regional lymph nodes increases both the risk of recurrence and mortality [49]. Furthermore, the five-year survival rate is shown to decrease by approximately 60% in patients with lymph node metastases [27]. The use of sentinel lymph node biopsy for regional staging in patients with cSCC remains a topic of ongoing debate [50,51,52,53]. For this reason, SLNB was included in our analysis despite the small number of patients who underwent the procedure (*n* = 8).

We did not observe a significant difference between the cohort with and without SLNB regarding locally recurrent tumors and the development of metastases. However, local recurrence was observed in 18.1% (*n* = 21) of patients who did not experience SLNB, whereas no local recurrences (0%) were reported in patients with SLNB (*p* = 0.349). Nevertheless, the small number of patients does not allow for any conclusion regarding the protective effect of SLNB and should be interpreted with caution. A recent study by Tejera-Vaquerizo et al. published in 2024 demonstrated a therapeutic benefit of SLNB in high-risk squamous cell carcinoma [2]: The authors conducted a retrospective, multicenter analysis comparing patients who underwent sentinel lymph node biopsy. Patients in the SLNB group had a 95% lower risk of nodal recurrence compared to those without SLNB, and disease-specific survival was improved, with an 83% reduction in disease-specific mortality [2]. Similar results with a better outcome in the case of SLNB being conducted compared to observation only were found by Zhang et al. [3]. Contrary results were published by Kofler et al., where no significant difference was observed between the SLNB group (11.9%) and the observation group (11.4%) (*p* = 0.873) [4].

Evidence remains inconclusive, and further research is needed. Larger prospective studies with standardized SLNB criteria, sufficient follow-up time and higher case numbers would be desirable to further investigate the benefits of sentinel lymph node biopsy in cSCC.

### 4.4. Metastases

In our study, a metastasis rate of 15.3% (*n* = 19) was observed, which is slightly higher compared to that found by Dinehart et al. (7.4%), Samsanavicius et al. (3.4%), Joseph et al. (4.9%) and Brantsch et al. (4%) [6,16,48,54]. In contrast to the patient cohort studied by Brantsch et al., our study only included patients with a tumor thickness greater than 3 mm, which could explain the higher metastasis rate.

In the present patient collective, it was demonstrated that the five-year survival rate was significantly higher in patients without metastases compared to those with metastases (83.9% versus 46.1%, ***p* = 0.036**). This underlines the results of a prospective cohort study of 210 patients by Schmults et al., showing that metastatic disease carries high mortality [55]. Knuutila et al. equally showed worse survival rates in metastatic cSCC [56].

## 5. Conclusions

The present study highlights the critical influence of tumor location, thickness and immunosuppression on the clinical outcomes of cutaneous squamous cell carcinoma (cSCC). While cSCC most frequently arises in UV-exposed skin, tumors on non-UV-exposed areas were thicker on average and associated with higher metastasis rates, possibly reflecting delayed detection. Most recurrences occurred from tumors in the head and neck region. Immunosuppression emerged as a critical risk factor, correlating with younger age at diagnosis, increased metastasis rates and reduced five-year overall survival. SLNB was only performed in only a small subset of patients; therefore it is not possible to draw generalizable conclusions. However, larger studies are required to confirm its efficacy in reducing recurrence and metastatic spreads. These findings emphasize the importance of individualized management approaches, particularly for high-risk patients, and highlight the need for future research to improve outcomes in cSCC.

## 6. Limitations

Several limitations should be acknowledged, including a limited number of patients and a relatively short follow-up period. As a retrospective, single-center study, our analysis is subject to inherent constraints, including potential selection and information biases. Patient inclusion was based on available documentation, which may have led to the underrepresentation of certain subgroups. Furthermore, the accuracy and completeness of clinical and histopathological data are contingent upon the quality of medical records, with certain variables such as additional histological details being inconsistently documented. These limitations may have influenced both the detection of associations and the generalizability of our findings.

In the future, it would be desirable to conduct further analyses with larger patient cohorts and to include additional histopathological features, such as tumor grading, desmoplasia and lymphovascular invasion.

Especially regarding SLNB, larger patient cohorts with clearly defined selection criteria are needed, as SLNB remains a widely debated topic in the management of cutaneous squamous cell carcinoma.

## Figures and Tables

**Table 1:** The baseline characteristics and tumor parameters of the study cohort.

Descriptive Analysis
**Gender *n* = 124** ○ Male *n* = 91 (73.4%) ○ Female *n* = 33 (26.6%)
**Age *n* = 124** ○ Median 79 years (IQR 72–85 years)
**Tumor location: *n* = 124** ○ Head and neck: *n* = 89 (71.8%) ○ *Capillitium: n = 24 (27.6%)* ○ *Ear: n = 14 (16.1%)* *Cheek: n = 10 (11.5%)* *Forehead: n = 10 (11.5%)* *Temple: n = 8 (9.2%)* *Nose: n = 6 (6.9%)* *Lips: n = 5 (5.7%)* *Neck: n = 2 (2.3%)* *Eyebrow: n = 2 (2.3%)* *Lower eyelid: n = 2 (2.3%)* *Other/Not specified: n = 6 (6.9%)* ○ Legs: *n* = 17 (13.7%) ○ Arms: *n* = 13 (10.5%) ○ Trunk: *n* = 5 (4.0%) **Tumor location UV-exposed skin:** ○ Yes: *n* = 113 (91.1%) ○ No: *n* = 11 (8.9%)
**Tumor thickness: *n* = 121 (3 missing)** ○ 3–5.9 mm: *n* = 64 (52.9%) ○ ≥6 mm: *n* = 57 (47.1%) **Tumor thickness by anatomical region:** ○ Trunk: Median 15.0 mm (IQR: 10.0–20.0 mm) ○ Head: Median 5.6 mm (IQR 3.6–7.6 mm) ○ Arms: Median 3.95 mm (IQR 2.0–6.95 mm) ○ Legs: Median 6.0 mm (IQR 3.75–8.25 mm) **Tumor thickness by UV-exposure:** ○ UV-exposed (head, neck, décolleté, forearms, hands, lower legs): 5.4 mm (IQR: 3.55–7.25 mm) ○ Non-UV-exposed (trunk, upper arms, thighs, feet): 8.0 mm (IQR: 6.0–10.0 mm) ○ ***p* = 0.001**
**Metastases *n* = 124** ○ Yes: *n* = 19 (15.3%) ○ No: *n* = 105 (84.7%) **Location of metastases:** ○ Lymph nodes: *n* = 17 (13.7%) ○ Cutaneous: *n* = 3 (2.4%) ○ Distant: *n* = 4 (3.2%) **Metastasis rate by UV exposure** ○ UV-exposed: 10.6% (*n* = 12) ○ Non-UV-exposed: 63.6% (*n* = 7) ○ ***p* < 0.001** **Metastasis rate by location:** ○ head and neck: 11.2% (*n* = 10) ○ trunk: 40.0% (*n* = 2) ○ arms: 15.4% (*n* = 2) ○ legs: 29.4% (*n* = 5) ○ *p* = 0.107 **Metastasis rate by tumor thickness:** ○ 3.0–5.9 mm: 3.1% (*n* = 2) ○ ≥6 mm: 26.5% (*n* = 15) ○ ***p* < 0.001**
**Local recurrence *n* = 124:** ○ Yes: *n* = 21 (16.9%) ○ No: *n* = 103 (83.1%) **Recurrence rate by location:** ○ Legs: 23.5% (*n* = 4) ○ Head and neck: 16.9% (*n* = 15) ○ Arms: 15.4% (*n* = 2) **Recurrence rate in relation to re-excision:** ○ Re-excision: 25.0% (*n* = 15) ○ No re-excision: 6.7% (*n* = 4) ○ ***p* = 0.006**
**Perineural invasion** ○ Yes (*n* = 4) ○ No (*n* = 119) ○ Missing (*n* = 1) **Perineural invasion and metastasis rate** ○ Yes: 25.0% (*n* = 1) ○ No: 15.1% (*n* = 18) ○ *p* = 0.49 **Perineural invasion and recurrence rate:** ○ Perineural invasion: 75.0% (*n* = 3) ○ No perineural invasion: 14.3% (*n* = 17) ○ ***p* = 0.013**

**Table 2:** Patient characteristics in relation to immunosuppression and clinical outcomes.

Variable	Immunosuppressed (*n* = 31)	Non-Immunosuppressed (*n* = 93)	*p*-Value
**Median age (years)**	74 (IQR 70.5–77.5)	81 (IQR 75.5–86.5)	**<0.001**
**Male sex**	64.5% (*n* = 20)	76.3% (*n* = 71)	0.222
**Cause for immunosuppression**	Cause for immunosuppression: ○ Organ transplant recipients: *n* = 8 (25.8%) ○ Chronic lymphocytic leukemia: *n* = 9 (29.9%) ○ Myeloproliferative disorder: *n* = 4 (12.9%) ○ Non-Hodgkin lymphoma: *n* = 3 (9.7%) ○ HIV infection: *n* = 1 (3.2%) Treatment with immunosuppressive therapy for other reasons (glucocorticoids, methotrexate): *n* = 6 (19.4%)		—
**Multiple cSCCs**	38.7% (*n* = 12)	24.7% (*n* = 23)	0.186
**Tumor on UV-exposed site**	83.9% (*n* = 26)	91.1% (*n* = 113)	0.159
**Median tumor thickness (mm)**	5.6	5.6	—
**Local recurrence rate**	25.8% (*n* = 8)	14.0% (*n* = 13)	0.166
**Metastasis rate**	29.0% (*n* = 9)	10.8% (*n* = 10)	**0.021**
**Mean follow-up duration (months)**	24.8	14.97	**0.013**

## Data Availability

Data are contained within the article.
